# Aging clocks: disrupted circadian rhythms

**DOI:** 10.18632/aging.101642

**Published:** 2018-11-13

**Authors:** Aiste Steponenaite, Stephany M. Biello, Gurprit S. Lall

**Affiliations:** 1 Medway School of Pharmacy, University of Kent, Chatham, UK; 2 Department of Psychology, University of Glasgow, Glasgow, UK

**Keywords:** aging, circadian rhythm, NMDA, NR2B, glutamate, light, circadian entrainment

The earth’s rotation around its very own axis generates the phenomenon that we refer to as our day and night. This change in environmental lighting has been an essential component of life; where throughout the evolutionary chain, organisms have used this signal as a timing cue to which they regulate/ synchronise their sleep-wake activity. It is this entrainment of both physiological processes and behavioural traits that define and, in some aspects, govern an individual’s life style through circadian linked routines. Mammals, are no exception to this rule, often being referred to as ’creatures of habit’. From inception through to old age humans tend to gravitate to structured and defined periodic sleep- wake patterns; an uncanny system innately programmed into every individual, contributing to good health and wellbeing. However, the caveat lies within the aging process itself. Aging of the physiological components that govern and maintain circadian rhythms in mammals result in disruption to the clock leading to problems in sleep, cognition and social function, to name but a few. Little is known regarding the underlying mechanisms driving such changes; thus, it is imperative that we understand the natural biological aging process so to develop therapies and treatments that improve the quality of life in our, ever growing, aging population.

Light is the most important factor in driving an entrained circadian rhythm, contributing significantly to stable sleeping routines- an aspect that, most commonly, deteriorates through aging [1]. In mammals, light signals are decoded by a subset of retinal ganglion cells within the eye, which in turn relay directly to the circadian clock located in the brain within a region termed the suprachiasmatic nucleus (SCN). The excitatory neurotransmitter glutamate is released onto the SCN and through a, predominantly, N-methyl-D-aspartate (NMDA) receptor driven cellular pathway, the presence of light is notified to the clock. The resulting cellular increase in Ca^2+^ further activates a number of intracellular signalling cascades, altering traditional clock genes expression and ultimately driving the output of a robust behavioural rhythm that is synchronised to the given lighting regime. It can be clearly seen that this is a pathway with multiple components and signal transduction routes. Aging, on the whole, impacts negatively on physiological function and there has been strong prior evidence to suggest age related changes within the SCN. Through senescence, SCN cellular activity alters with decreases in glucose uptake and declines in oscillatory activity combined with dampening amplitudes of SCN neurons [2,3].

**Figure 1:**
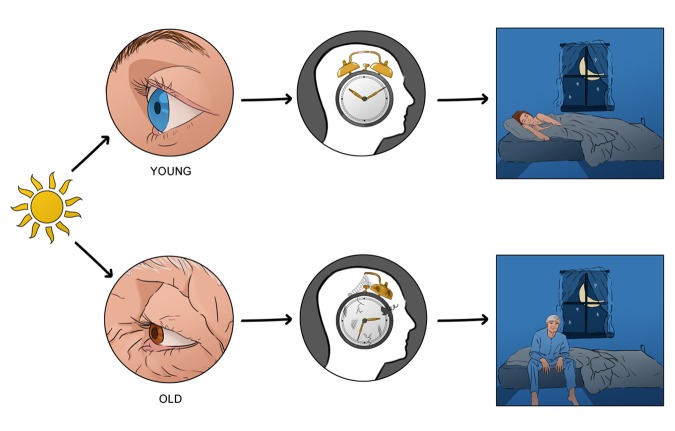
**Light input to the circadian clock is impaired through age induced cellular changes in the circadian clock- impacting entrainment of sleep wake rhythms.** Light is decoded by the eye; however little changes are seen in the aged eye with respects to input into the circadian clock. In fact, it is the clock itself that displays a decline in its ability to interpret this information, through alterations in glutamatergic signalling via the NMDA receptor; which ultimately result in global physiological desynchronization leading to implications in health and wellbeing.

These studies provide little insight into the light driven changes that may occur through aging, an important component for circadian entrainment. Thus, we set out to try and understand just this: how does light interpretation by the circadian system change with age, if at all [4]? Our initial findings showed striking differences in the effects of light to alter behavioural daily rhythms. In older animals, light resetting was significantly reduced, at both dim and bright intensities. In order to pinpoint the entities responsible for this deficit, we began by systematically investigating potential elements of the light cascade that may contribute to this observed decline in behavioural output. The visual system presents as the initial candidate. Conditions such as cataracts and/or alterations in cell morphology or death due to disease all impact the transmission of light. Looking at the light sensitivity of the aging eye, we found that to bright light there was no significant differences in decoding between young and old mice. It is to these bright intensities that the circadian system responds. Taken together, these results suggested that any physiological alterations lay at the level of the SCN. Both *in vivo* and *in vitro* administration of NMDA, a glutamatergic agonist, resulted in reduced clock resetting in aged animals when compared to their younger counterparts. This allowed us to hone in on our target, as it identified glutamate as the culprit through actions via the NMDA receptor. On closer investigation, the NMDA receptor has been linked to aging events. In particular the NR2B subunit of this receptor was found to decrease in expression in cortical regions and dentate granule cells of aged mouse brains [5]. Data from our studies also found that the NR2B subunit exhibited changes within the SCN, presenting with a decrease in expression in aged mice. This structural alteration in the NMDA receptor configuration in the old animals was, most likely, the reason for the decrease in light input to the SCN. To further cement our findings and conclusion, we administered a NR2B antagonist to younger mice and were able to generate the phenotypic response observed in older mammals to light (Figure 1).

The signs of aging manifest in many different ways, be it a slow decline in physical abilities through to increased vulnerability to disease [6]. With a rapidly growing aging population worldwide, it is not enough to just focus on managing disease; there is a need for us to understand the natural aging process and the mechanics that underlie age related changes, to better provide preventative medical interventions and promote lifelong wellbeing.
